# Low Iodine Intake May Decrease Women’s Fecundity: A Population-Based Cross-Sectional Study

**DOI:** 10.3390/nu13093056

**Published:** 2021-08-31

**Authors:** Mingluan Xing, Simeng Gu, Xiaofeng Wang, Guangming Mao, Zhe Mo, Xiaoming Lou, Xueqing Li, Xuemin Huang, Yuanyang Wang, Zhifang Wang

**Affiliations:** Department of Environmental Health, Zhejiang Provincial Center for Disease Control and Prevention, Hangzhou 310051, China; mlxing@cdc.zj.cn (M.X.); smgu@cdc.zj.cn (S.G.); xfwang@cdc.zj.cn (X.W.); gmmao@cdc.zj.cn (G.M.); zhmo@cdc.zj.cn (Z.M.); xmlou@cdc.zj.cn (X.L.); xqli@cdc.zj.cn (X.L.); xmhuang@cdc.zj.cn (X.H.); yywang@cdc.zj.cn (Y.W.)

**Keywords:** iodine deficiency, time to pregnancy, fecundity, fecundability ratio

## Abstract

Salt iodization is one of the most cost-effective strategies to eliminate iodine deficiency disorders (IDD). However, China’s dismantling of salt monopoly has reduced the availability of iodized salt in the susceptible population in pregnancy, which might cause IDD and have adverse health effects on both themselves and their offspring. The aim of our study was therefore to explore the association between IDD and women’s reproductive health. This is a population-based cross-sectional study conducted in 2018 in Zhejiang Province, China. A total of 1653 pregnant women participated in this study. Median urinary iodine concentration (UIC) in the population was used to assess iodine intake. Cox regression analyses were used to estimate the association between iodine intake and time to pregnancy, which was indicated with fecundability ratio (FR) and 95% confidence interval (CI). The percentage of participants with iodine deficiency who had been waiting longer than 13 months to get pregnant (20%; median UIC 119.6 μg/L) was significantly higher than those with iodine sufficiency (14%; median UIC 147.1 μg/L). A significant decrease in fecundity was observed in participants with iodine deficiency (FR, 0.820; 95% CI, 0.725−0.929) than those with iodine sufficiency. These findings indicate the importance of ongoing monitoring of iodine nutrition in women of reproductive age. Keeping a safe and optimal level of iodine nutrition during pregnancy should be emphasized.

## 1. Introduction

Salt iodization is one of the most cost-effective strategies to eliminate iodine deficiency disorders (IDD). China, a historically severely iodine-deficient country, introduced the universal salt iodization (USI) program in 1995, with a legislative requirement for the addition of iodine in edible salt. Further, the requirement stated that non-iodized salt for household cooking must be sold only at designated places via a physician’s prescription. The goal of IDD elimination at a country level has been achieved since 2000. However, in some parts of China where increasing incidence of thyroid diseases were reported with increasing iodine intake [1,2,3,4,5], awareness was raised about the possible association between consumption of iodized salt and the escalating incidence of thyroid diseases [1,6,7]. An increasing number of medical practitioners and the public began to lose confidence about the consumption of iodized salt.

When the salt monopoly was dismantled in 2016, the population, especially those residing in Zhejiang Province where many salt factories are located, could easily access non-iodized salt [8]. Wide distribution of non-iodized salt can reduce iodine intake in the population and present hazards to sustainable IDD elimination, as shown in the recent provincial-level surveillances for IDD, especially among the pregnant population [9,10]. When the coverage of household iodized salt was more than 95%, the median UIC of pregnant women was 148.72 μg/L [9], suggesting marginal iodine sufficiency according to WHO’s assessment criteria of the lower cut-off value of optimal iodine intake (150 μg/L). When the coverage dropped to nearly 80%, the median decreased to 130.47 μg/L [10], implying mild iodine deficiency based on the assessment by WHO and previous published studies [11,12,13].

The public health problem associated with iodine deficiency in pregnancy occurs in both developed [14,15,16] and developing countries [11,17,18] due to the role iodine plays in thyroid health [2,19,20] and neurodevelopment of the fetus [12,14,21]. Iodine is a nutritional element, whose impact on women’s fertility is often overlooked. It is known that severe IDD poses a significant risk to reproduction, including spontaneous abortion, stillbirth, premature birth, and a decrease in fecundability [22,23,24,25]. However, there is limited knowledge concerning the damage to women’s reproductive health, particularly fertility, due to mild IDD. The present study aimed to understand the association between mild iodine deficiency and women’s reproductive abnormalities, mainly focusing on fecundability ratio (FR), which are the odds of conception.

## 2. Materials and Methods

### 2.1. Study Design and Study Population

Data concerning urinary iodine concentration (UIC) of pregnant women between 2015 and 2017 were drawn from the Zhejiang Electronic Surveillance Reporting System. Iodine intake was assessed according to the criteria of the World Health Organization (Iodine sufficiency: 150−249 μg/L; Iodine deficiency: <150 μg/L) [26]. Median UIC was used to estimate population-level iodine intake, due to the lack of an effective assessment indicator for individuals [26].

From March to December 2018, a cross-sectional retrospective study was conducted among pregnant women participating in the Zhejiang surveillance on IDD from the coastal regions (Zhoushan, Ningbo, Wenzhou, and Taizhou cities) and inland regions (Jinhua, Quzhou, and Lishui cities). The study aimed to collect data about the time required to conceive (time required for the current pregnancy). The sampling method of the IDD surveillance was comprehensively reported in our previous studies [10,27]. In brief, both the coastal and the inland regions were first selected. Secondly, 59 county-level administrative divisions were selected from the abovementioned seven cities. Third, five towns from each county were selected according to special locations. Finally, 20 pregnant women were selected from each selected town. At community healthcare centers, 6126 married pregnant women in any trimester who had been living in the local areas for at least one year were enrolled. The women were required to provide samples of spot urine and household cooking salt to determine UIC and salt iodine concentration. No significant difference was observed in the percentage of participants who had planned the current pregnancy, with 31.2% (1121/3598) from the coastal regions vs. 32.8% (830/2528; *p* = 0.166) from the inland regions (Supplementary Table S1).

In the present study, the trained obstetrician had a face-to-face interview with each participant to complete a paper-based questionnaire relating to the assessment of time to the current pregnancy. Consensus regarding the questionnaire, which was adapted from previous studies [4,5,6] and adopted after the agreement, was reached by three subject experts. The questionnaire was comprised of two sections. For the first section, participants were asked whether they had received the assistance of any fertility treatment to conceive. If the participants answered negatively, they were asked whether they had planned their pregnancy. If the participants answered positively, they were asked about the date of stopping the use of contraceptives to active conception and the end date of conception, which was determined as the 1st day of the last menstrual period. If needed, the 1st day of the last menstrual period was drawn from the national electronic prenatal record system. Time required to conceive was recorded as number of months, and was calculated as the number of months between the dates of stopping the use of contraceptives to the end point of conception. The second section was related to the potential confounders, which were considered according to knowledge of literature [28] and empirical evidence including: date of birth, ethnic group, post code, highest educational qualification, occupation, income per capita in 2017, gestational age, gravidity, prepregnancy weight and height, history of thyroid diseases, and history of spontaneous abortion if multigravida pregnancy was reported. Prepregnancy BMI was calculated using the following formula: BMI = weight/height^2^ (kg/m^2^). History of spontaneous abortion was defined as the loss of pregnancy at less than 20 weeks of gestation in the absence of elective medical or surgical measures to terminate the pregnancy.

**Figure 1:**
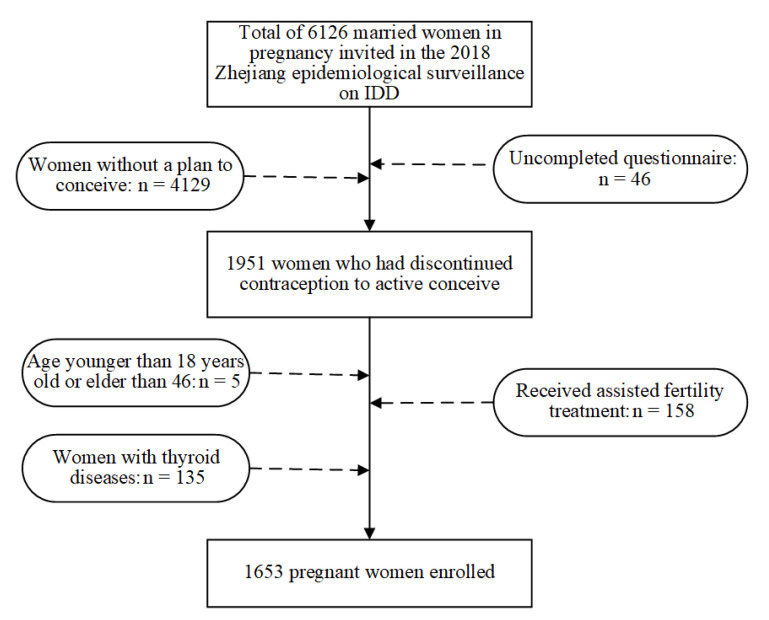
Study flowchart illustrating the study design.

The inclusion criteria for the study included women aged 18.0 and 46.0 years old at enrollment, who had self-reported discontinuing contraceptive use to conceive, had planned the current pregnancy, women with multigravida, and those who were able to communicate. Women who self-reported having received assisted fertility treatment, either partner had sought any medical help to get pregnant, or those who had a history of thyroid diseases were excluded from the study. A flowchart of the procedure of exclusions for the study participants is shown in Figure 1.

### 2.2. Statistical Analyses

R statistical software (version 4.0.2, R Core Team, Vienna, Australia) was performed. Packages of both survival (https://cran.r-project.org/web/packages/survival/index.html, accessed on 28 July 2021) and survminer (https://cran.r-project.org/web/packages/survminer/index.html, accessed on 28 July 2021) were used to compare time to pregnancy between the two groups [23]. Count data were expressed as numbers and percentages (%). Continuous data were described via mean and standard deviation (SD). Between groups, comparisons of the count data were performed via chi-square tests while comparisons of the continuous data were performed via t-tests. Kaplan–Meier survival curves were performed to record the number of months required to conceive for each group. To assess the effects of iodine deficiency concerning the months required to conceive, months required to conceive were censored at 13 months and above according to the WHO’s clinical definition of infertility [29]. The log-rank test was used to determine if distribution of time duration significantly differed between two groups. Cox proportional hazards regression models were used to investigate the association between time required to conceive and iodine inadequacy, and were indicated with FRs and 95% confidence intervals (CI) before and after the adjustment of covariables. The FR represented the monthly probabilities of conception in the iodine-deficient participants relative to a reference of the iodine-sufficient participants. An FR less than 1 indicated reduced fecundity.

## 3. Results

### 3.1. Characteristics of the Total Studying Participants

Note that 1653 eligible participants were included for the final analyses, 958 from the coastal regions vs. 695 from the inland regions. The median UIC of 958 participants from the coastal regions was 119.6 μg/L (95% CI 113.4−124.0 μg/L), while the median UIC of 695 participants from the inland regions was 147.1 μg/L (95% CI 144.0−153.0 μg/L). The observed media UIC indicated that these samples were sufficiently representative to estimate iodine intake, which was consistent with the regional epidemiological surveillance results between 2015 and 2017 (Supplementary Table S2). Coverage of household iodized salt was 70.3% (673/958) for participants from the coastal regions and 96.1% (668/695) for participants from the inland regions.

Table 1 shows the characteristics of the study participants compared by iodine intake. When compared with iodine-sufficient participants, a greater number of iodized-deficient participants were young (*p* < 0.001), of Han ethnicity (*p* < 0.001), office workers (*p* < 0.05), primigravida (*p* < 0.05), with high educational qualifications (*p* < 0.001), and high income (*p* < 0.001). No differences in the prepregnancy BMI and history of spontaneous abortion were defined between these two groups (both *p* > 0.05).

### 3.2. Time to Pregnancy

**Figure 2:**
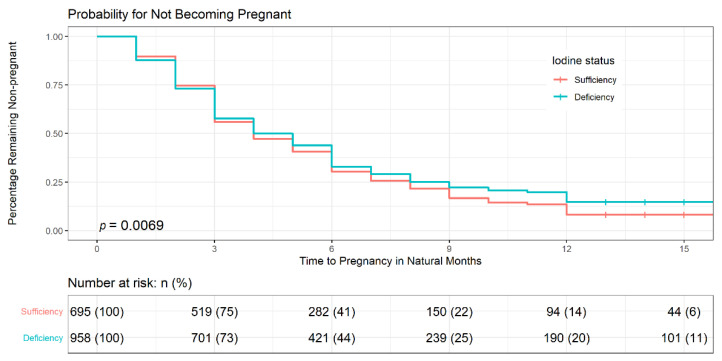
Distribution of time to pregnancy by iodine intake via Kaplan–Meier survival curves. Log-rank test was used to assess the difference in time to conceive between participants with iodine sufficiency and iodine deficiency (*p* = 0.007). Kaplan–Meier survival estimates showed that participants with iodine deficiency took a longer time to get pregnant than those with iodine-sufficiency. +: censoring times.

Distribution of time to pregnancy was estimated according to iodine intake via Kaplan-Meier survival curves (Figure 2). The median time to get pregnant was longer for participants with iodine deficiency (5 months) than those with iodine sufficiency (4 months), though no significant differences were observed (*p* = 0.266). The log-rank test showed that the percentage of iodine-deficient participants who failed to get pregnant after waiting for at least 13 months was significantly higher (20%) than those with iodine-sufficiency (14%; *p* = 0.007).

To further explore the association between iodine inadequacy and FR, we performed Cox regression analysis. The results, before adjustment of covariables, showed that compared to participants having iodine sufficiency, iodine-deficient participants were significantly less likely to get pregnant (unadjusted FR = 0.814, 95% CI 0.718−0.923). After the adjustment of the potentially significant covariables (age, prepregnancy BMI, income, occupation, education, and history of spontaneous abortion; Supplementary Table S3), Cox proportion hazard regression analyses showed that iodine inadequacy had significantly adverse effects on FR (Model 5: adjusted FR 0.820, 95% CI 0.725−0.929; Table 2). The result remained robust when the analyses were expanded to include primigravida, women with thyroid diseases, those receiving treatment for infertility, those aged less than 18 and older than 46 years, and all pregnant women who planned their current pregnancy (Table 2). Based on our model, the fecundability ratio decreased by 19.4% among iodine-deficient women aged 30.5 years, with average BMI 21.7 kg/m^2^, education level higher than 14 years, having domestic workers, and no history of spontaneous abortion, compared with similar women with iodine sufficiency (Supplementary Figure S1).

## 4. Discussion

Though Zhejiang has achieved IDD elimination in pregnancy at a provincial level since 2011 [30], iodine status is regionally heterogeneous at a sub-provincial level, with iodine deficiency in coastal regions and iodine sufficiency in inland regions [27,31], presenting a unique setting throughout China to investigate the association between iodine deficiency and time required to conceive. Iodized salt accounted for 70–80% of the dietary iodine source for Zhejiang’s women [32]. In the present study, the percentage of iodine-deficient participants using iodized salt was significantly lower than that of participants having iodine sufficiency (86.43% vs. 97.55%). Therefore, inadequate iodine intake in coastal population may attribute to low coverage of household iodized salt (70.3%), which is below the Chinese criteria of elimination of IDD of 95% [33], when compared with 96.1% of the participants from the inland region with sufficient iodine intake.

Important associations between iodine intake and demographic characteristics were identified in this study. We found that younger, better-educated women and white-collar workers were more likely to have a UIC of less than 150 μg/L. Similar results have been reported in Zhejiang Province [34] and Shanghai [35]. This could be explained by participants’ awareness and behaviors towards iodine nutrition. To be specific, elder women tend to have a better-quality diet, which plays an active role in iodine intake [36]. It should be mentioned that some studies reported that an increased incidence of thyroid diseases was considered to be associated with a high intake of iodized salt in coastal provinces [2,37,38]. These results were done by physicians according to medical records information rather than a representative population. As a result, Berkson’s bias seems inevitable and the incidence of thyroid diseases was exaggerated. It is easy to make people feel confused even though they had a high level of education. Some individuals believed that the population in the coastal area would have an excessive iodine intake based on daily consumption of seafood and iodized salt should be limited. In addition, all of the above cofounders we chose are typical of those that might be predisposed for the risk of prolonged time to pregnancy [39,40].

This is the first study to investigate the effect of iodine deficiency on the time required to conceive among women in China. Our findings showed that women with the median UIC of 119.6 μg/L (95% CI 113.4−126.0 μg/L) from the coastal regions had a reduced percentage of FR (19.4%) over a natural month in comparison to those women with the median value of 144.1 μg/L (95% CI 137.0−151.0 μg/L) from the inland regions. It indicated that mildly iodine-deficient women required a significantly longer time to get pregnant than women with iodine sufficiency. It is consistent with the results of a prospective cohort study that women with severe iodine deficiency had a lower chance of becoming pregnant when compared with those with normal iodine intake [22].

Some biological mechanisms may account for these findings. The body does not synthesize iodine, which is an essential nutrient for the synthesis of thyroid hormones and hence needs iodine supplementation via dietary sources. Insufficient iodine intake leads to hypothyroidism, which has adverse impacts on folliculogenesis, ovulation, and maturation of the corpus luteum, and ultimately influences fertility [41]. Hypothyroidism in adult non-pregnant women is common in China [37,42] and needs urgent attention.

China has adopted the universal two-child policy since 2015, with the relaxation of one-child policy, which has been implemented for more than three decades. Under the new circumstances, couples had the option of family planning. In this study, the most recent pregnancy was recalled, and more than 90% pregnancies were in the last 1.5 years. It has been documented that time about the recent pregnancy, which was recalled via a questionnaire, bears a similarity in both validity and reliability to prospective time to pregnancy [43,44,45]. Thus, recall bias is minimal in this study. In addition, iodine intake was assessed in the general population rather than individuals [26]. Further, residents with iodine deficiency or iodine sufficiency were unlikely to have been misclassified since regional variety of iodine intake in this study is in agreement with previous surveillance [27,46].

There are some limitations that need to be noted. First, data concerning the iodine intake in the population comprising women of reproductive age are not available since iodine intake in the susceptible population has focused on both pregnant women and school-age children in the current national IDD surveillance system. Since borderline thyroid deficiency may be linked to women’s infertility [34], monitoring of the iodine intake of women of reproductive age should be strengthened in future. Second, the majority of the study participants were of Han ethnicity. Our findings may not be explained among the other minor groups. Third, talking about sexual matters in the public realm remains a taboo topic in China. It is a great challenge to collect data concerning sexual behavior, for example, frequency of intercourse. Four, the study design was retrospective, which limited the potential for checking the data accuracy or to consider the temporal relationships. For example, it might be possible that some women start to use iodine supplementation during pregnancy, raising their iodine intake and possibly changing their iodine intake category from the pre-pregnancy to pregnancy period. Five, for residents living in the coastal area, seafood is an essential component of their daily dietary intake. It might be instructive to assess the impact of eating habits on UIC as well. Previous studies indicated that seafood is a minor dietary iodine source, contributing to about 10% of daily iodine intake, while iodized salt is the main source of dietary iodine. It contributes to at least 70% of daily dietary intake [32,47]. Taken together, further studies with prospective design, diversity of population and detailed information are warranted to validate the above findings and elucidate the corresponding biological mechanism.

## 5. Conclusions

Our study showed that women with mild iodine deficiency showed a positive association with risk of prolonged time to pregnancy. With wide accessibility of non-iodized salt in China, there are a growing number of women with low iodine intake. Thus, ongoing monitoring of the population iodine status and education intervention should be considered. Further studies with more detailed information, such as dietary iodine habits, are warranted to validate the above findings and elucidate the underlying regional differences in iodine nutrition.

## Figures and Tables

**Table 1:** Characteristics of the study participants compared by iodine intake.

Variables	Iodine Sufficiency *n* (%)	Iodine Deficiency *n* (%)	*p*
Age (years)	30.2 ± 5.0	29.0 ± 4.3	<0.001
Age group (years)			<0.001
<30	360 (51.8)	593 (61.9)	
≥30	335 (48.2)	365 (38.1)	
Ethnic group			<0.001
Han	651 (93.7)	936 (97.7)	
Others	44 (6.3)	22 (2.3)	
Prepregnancy BMI			0.378
≤18.4	104 (15.0)	155 (16.2)	
18.5–23.9	465 (66.9)	653 (68.2)	
≥24.0	126 (18.1)	150 (15.7)	
Education (years)			<0.001
≤9	240 (34.5)	255 (26.6)	
≥10	455 (65.5)	703 (73.4)	
Income per capita (USD)			<0.001
<10,000	346 (49.8)	363 (37.9)	
10,000–15,999	204 (29.4)	310 (32.4)	
≥16,000	145 (20.9)	285 (29.7)	
Occupation			0.001
Office workers	324 (46.6)	487 (50.8)	
Domestic workers	244 (35.1)	359 (37.5)	
Others	127 (18.3)	112 (11.7)	
Primigravida			0.010
Yes	172 (24.7)	292 (30.5)	
No	523 (75.3)	666 (69.5)	
Spontaneous abortion history			0.750
Yes	29 (4.2)	37 (3.9)	
No	666 (95.8)	921 (96.1)	
Sum	695 (100.0)	958 (100.0)	

BMI, body mass index; Chi-square tests were used when percentages were compared between groups of two and above; *t*-tests were performed when age was compared between two groups.

**Table 2:** The associations between iodine inadequacy and adjusted fecundability ratios via Cox regression analyses.

Model	Iodine Intake	*n*	Adjusted Fecundability Ratio ^&^ (95% CI)	*p*
Model 1: All women having a plan for this current pregnancy (*n* = 1951)				
	Sufficiency	830	1	
	Deficiency	1121	0.809 (0.734−0.893)	<0.001
Model 2: Excluding women aged less than 18 years old and elder than 46 (*n* = 1946)				
	Sufficiency	826	1	
	Deficiency	1120	0.810 (0.734−0.893)	<0.001
Model 3: Excluding women using received treatment for infertility (*n* = 1788)				
	Sufficiency	754	1	
	Deficiency	1034	0.798 (0.721−0.883)	<0.001
Model 4: Excluding women with thyroid diseases (*n* = 1653)				
	Sufficiency	695	1	
	Deficiency	958	0.823 (0.741−0.914)	<0.001
Model 5: Excluding women with primigravida (*n* = 1189)				
	Sufficiency	523	1	
	Deficiency	666	0.820 (0.725−0.929)	<0.001

^&^: Adjusted for age, prepregnancy BMI, education, income, occupation, and spontaneous abortion history.

## Supplementary Materials

The following are available online at https://www.mdpi.com/article/10.3390/nu13093056/s1, Table S1: Demographic characteristics among the total of 6126 pregnant women, by whether they reported to have their plan for the current pregnancy. Table S2: Iodine intake in population in pregnancy in Zhejiang between the coastal and inland regions, 2015–2017. Table S3: The association between potential covariates and fecundability ratios via univariate Cox regression analyses. Figure S1: Cox regression analyses for iodine inadequacy and adjusted fecundability ratios.
