# Supplementary material for: Low Iodine Intake May Decrease Women’s Fecundity: A Population-Based Cross-Sectional Study
Source: Nutrients. 2021 Aug 31;13(9):3056. doi: 10.3390/nu13093056 (PMC8467427; doi:10.3390/nu13093056)
Supplement: Supplementary file 1 [file nutrients-13-03056-s001.zip › nutrients-1339045-supplementary.pdf]

Table S1. Demographic characteristics among the total of 6126 pregnant women, by whether they reported to have their plan for the current pregnancy

Variables	Non-planners (N=4175)	Planners (N=1951)
Iodine status		
Deficiency	59.3	57.5
Sufficiency	40.7	42.5
Age (years)	28.9±5.3	29.6±4.8
Age group (years)		
< 30	56.3	62.3
≥ 30	43.7	37.7
Ethnic group		
Han	95.2	96.2
Non-Han	4.8	3.8
Prepregnancy BMI		
≤ 18.4	16.0	15.3
18.5–23.9	66.5	67.2
24.0–27.9	14.4	13.2
≥ 28.0	3.1	4.3
Education		
≤ 9 years	40.0	30.4
10–13 years	23.8	20.9
≥14 years	36.2	48.7
Income per capita (USD)		
<10,000	50.6	42.6
10,000 –15,999	31.1	31.5
≥ 16,000	18.3	25.9
Occupation		
Office workers	42.4	49.3
Domestic workers	37.3	36.0
Others	20.3	14.7
Primigravida		
Yes	28.9	27.9
No	71.1	72.1
History of spontaneous abortion		
Yes	76.5	77.4
No	23.5	22.6

Table S2. Iodine intake in population in pregnancy in Zhejiang between the coastal and inland regions, 2015–2017

Region	Year	N	Median UIC (95% CI), μg/L	Iodine status[¶]
Coast	2015	3369	127.0 (122.4–130.0)	Deficiency
	2016	3286	119.2 (116.6–123.1)	Deficiency
	2017	3507	113.5 (110.0–116.4)	Deficiency
Inland	2015	1952	152.5 (147.7–155.4)	Sufficiency
	2016	2226	146.0 (140.5–152.0)	Sufficiency
	2017	2469	154.0 (150.0–156.9)	Sufficiency

[¶]Iodine status in population in pregnancy was assessed based on the WHO criteria of optimal iodine of 150–249 μg/L.

Table S3. The association between potential covariates and fecundability ratios via univariate Cox regressions analyses

Variable	N	Fecundability ratio (95% CI)	*p*
Iodine nutrition group			
Iodine sufficiency	695	1	
Iodine deficiency	958	0.814 (0.718–0.923)	0.001*
Age group			
< 30 years	700	1	
≥ 30 years	953	0.725 (0.657–0.810)	< 0.001**
Prepregnancy BMI			
18.5–23.9	1118	1	
≤ 18.4	259	1.013 (0.839–1.224)	0.892
≥ 24.0	276	0.846 (0.721–0.993)	0.040*
Spontaneous abortion			
No	1587	1	
Yes	66	0.735 (0.558–0.968)	0.029*
Primigravida			
Yes	464	1	
No	1189	1.095 (0.976–1.227)	0.1
Ethnicity			
Others	66	1	
Han group	1587	0.782 (0.605–1.011)	0.070
Occupation			
Office workers	811	1	
Domestic workers	603	0.859 (0.745–0.990)	0.036*
Others	239	0.901 (0.745–1.089)	0.280
Education (years)			
≤ 9	811	1	
10–13	603	1.076 (0.930–1.244)	0.324
≥14	239	1.231(1.092–1.389)	<0.001**
Income (USD)			
<10,000	709	1	
10,000–15,999	514	1.066 (0.921–1.233)	0.393
≥ 16,000	430	1.223 (1.040–1.438)	0.015*

* $p < 0.05$; ** $p < 0.001$.

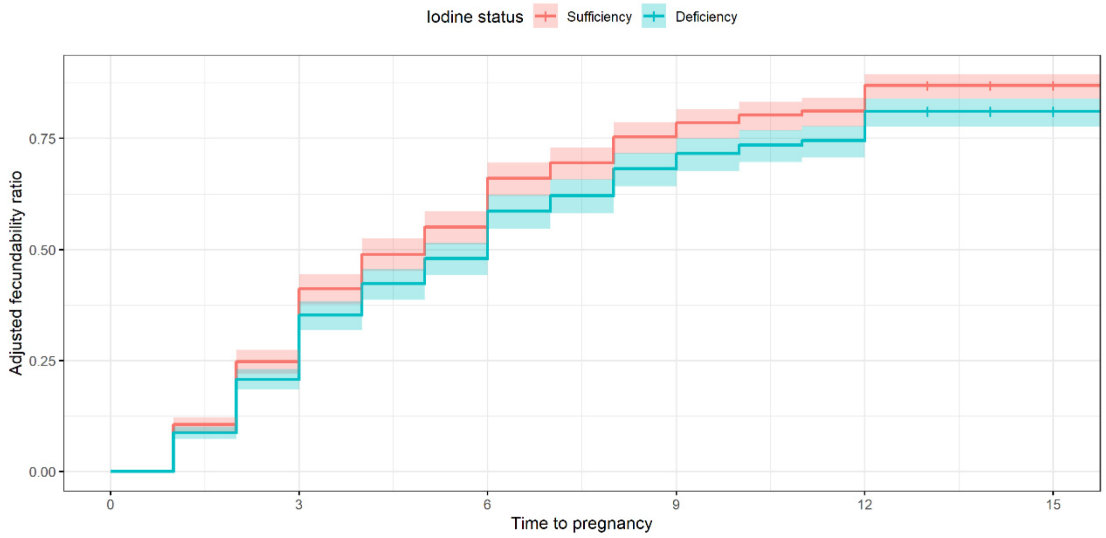

Figure S1: Cox regression analyses for iodine deficiency and adjusted fecundability ratios.
